# A Short-Term Evaluation of Foot Pronation Tendency in Healthy Recreational Runners

**DOI:** 10.3390/life13112202

**Published:** 2023-11-12

**Authors:** María José Galloso-Lagos, María Luisa González-Elena, Ana Juana Pérez-Belloso, Manuel Coheña-Jiménez, Mar Elena-Pérez, Juan Manuel Muriel-Sánchez, Aurora Castro-Méndez

**Affiliations:** 1 Podiatry Department, University of Seville, 41009 Seville, Spain; mariajgalloso98@hotmail.com (M.J.G.-L.); maruchi1@us.es (M.L.G.-E.); aperez30@us.es (A.J.P.-B.); mcohena@us.es (M.C.-J.); 2 Departamento de Ingeniería Electrónica, Universidad de Sevilla, 41092 Sevilla, Spain; marelen@us.es; 3 Independent Researcher, Clínica Centro Lepe, Calle Rincona, 31, 21440 Lepe, Spain; murielsanchezjm@gmail.com

**Keywords:** pronation, foot posture index, navicular drop, running

## Abstract

Running is a highly physical activity, and it is related to injuries when there is an excessive pronation of the foot. This study evaluates, from a sample group of healthy recreational runners, if the foot tends to pronate after a period of running activity and when, with respect to a period of running compared to walking, evaluated during several phases: after 30, 45, and 60 min. This quasi-experimental study has been carried out on a total of 36 healthy recreational subjects. The subjects were evaluated during two different activities: running activity for a period of an hour with respect to normal walking activity. The main outcome measures were the foot posture index (FPI) and the navicular drop test (NDT), which were evaluated at p1 (the screening day), after 30 min of activity (p2), after 45 min of activity (p3), and finally after 60 min (p4) during running or walking activity. The analysis showed significant differences for the FPI and NDT variables in both groups and on both feet, comparing p1 and p4. These changes showed a significant relationship comparing p1 and p3 for the FPI variable, and for the NDT variable (*p* < 0.001) of the left foot and, with respect to the right foot, significance was shown to the FPI comparing the p1 and p2. A significant difference was found in the tendency to pronate the foot after a period of running compared to the same period of walking after 60 min of activity. Running produced an excessive pronation of the foot after 45 min of activity, evaluated with the FPI for both feet.

## 1. Introduction

Running is a sport practised worldwide and seen as a healthy sport and is considered a highly physically demanding activity that requires great resistance from the structures that maintain the morphology of the foot [1,2,3,4,5,6,7,8]. Foot hyperpronation is defined as an excessive tendency to a foot posture index, which is related to the appearance of injuries in the foot and other levels of the musculoskeletal levels (fasciitis, ankle sprains, knee, or low back pain, for example) [1,2,3,4]. The internal longitudinal arch of the foot (MLA) is maintained by the passive action of the bone and ligamentous structures and by the plantar aponeurosis and, in turn, in an active way, due mainly to its extrinsic and intrinsic musculature [2]. The intrinsic foot muscles have an important role in the foot morphology and biomechanics that maintain the MLA.

It is considered relevant to identify risk factors that can alter the proper functioning of the foot to avoid injuries. Muscle fatigue can be correlated with excessive foot pronation. The collapse of the foot structures will induce a change in their morphology, tending to excessive foot pronation or hyperpronation that may be induced by muscle fatigue after a period of running activity, as has been evaluated in several previous researches [3,4,5,6,7,8].

These studies have analysed what appears to be the question of whether muscle fatigue is evaluated after a running period and may modify or induce foot pronation due to lack of support [2,5,6,7,8,9,10,11,12,13,14,15]. However, this question presents limited scientific evidence [15]. It is relevant to assess whether this physical activity induces muscle fatigue, assessed as a tendency to pronate, and at what time this occurs compared to walking with respect to running activity.

The implementation of muscular exercises has been associated with lower rates of injuries in runners and normalisation of walking and running, improving technique, and injury prevention [8,10,14,15,16,17,18]. Clinical tools such as the foot posture index (FPI) or the navicular drop test (NDT) can easily assess the effect of foot posture before and after the use of treatments that contribute to improving its functionality [11,12,19,20,21,22,23,24,25].

The originality of this work is based on the objective and focused assessment of a sample of non-pronating subjects: healthy adults who are not professional athletes. Also, if the FPI or ND is modified during different phases of the race for one hour compared to another activity, such as running, as a possible consequence of muscle fatigue in several short time intervals.

This research is justified due to the important role of identifying the suitability of implementing training to reduce muscle fatigue. Detecting situations and identifying when muscles become fatigued in healthy subjects can help implement strategies such as training to prevent muscle fatigue in the sports world. The main objective of this research was to determine whether there is muscle fatigue in healthy runners after an hour of running compared to a normal walk of the same period. 

Therefore, the null hypothesis of this study would be that the position of the foot or its pronation is not modified because of its musculature fatigue during a period of running; in contrast, the alternative hypothesis is that the foot tends to pronate during running compared to walking with respect to FPI and NDT.

## 2. Materials and Methods

A quasi-experimental longitudinal study was conducted on 36 healthy adults (n = 72 feet). The same subject sample was assigned to an experimental group (running activity) and a control group (walking activity) to minimise the risk of bias. An analysis of the study variables of repeated measurements (pronation tendency) was conducted for a total of seven times.

The ethical requirements required by the World Medical Association’s Ethics (Helsinki Declaration) experiments with human beings were respected [26]. Ethical approval was requested and granted by the Ethics Committee of the Virgen del Rocío y Macarena of the Seville Hospitals (Spain). Ethical approval code: 0973-N-20. Informed consent was obtained from all subjects involved in the study.

### 2.1. Participant Recruitment

A total of 36 participants attended the sports podiatry service of the University of Seville’s Podiatry Clinic Area (PCA) between September 2022 and June 2023. Informed consent was obtained from all subjects involved in the study, with each subject expressing voluntary participation in the study. There was no reward for participation or prejudice if they wished to leave the study. Those who voluntarily agreed to it and met the inclusion criteria were included.

The inclusion criteria were healthy, non-pronating adults of both sexes who came to the PCA as a patient or companion on the date indicated and were recreational runners dedicated to running at least three times a week for at least one hour. The exclusion criteria were an alteration or deformity that prevents a normal walk, pregnancy, ongoing rehabilitation treatment, or serious illness.

### 2.2. Procedures

The initial or baseline data (p1) were collected on the first day of participation in the research. Variables were sex, age, body mass index (BMI) (collected with an Adamson^®^ A23 model configuration scale), and height (Seca 222 stadiometer^®^). The identification of the dependent variables was performed during the baseline data analysis: the foot pronation tendency was measured with the foot posture index (FPI) and the navicular drop test (NDT). The biomechanical analysis of the FPI and NDT was taken for both feet on the day indicated for this, and the subject had to attend without fatigue or having performed any physical activity that day (a date before the race/experimental activity).

The participants were asked to carry out two activities: one considered the experimental (running activity) was to run for one hour at a comfortable speed selected as habitual for them in their activity and adequately equipped according to their comfort criteria (sneakers and adequate equipment for running); the other activity used as a control in this study was the same period of normal walking activity (comfortable speed chosen by the subject). An experienced podiatrist attended all subjects, collected data on all research variables and conducted a biomechanical examination.

**Figure 1:**
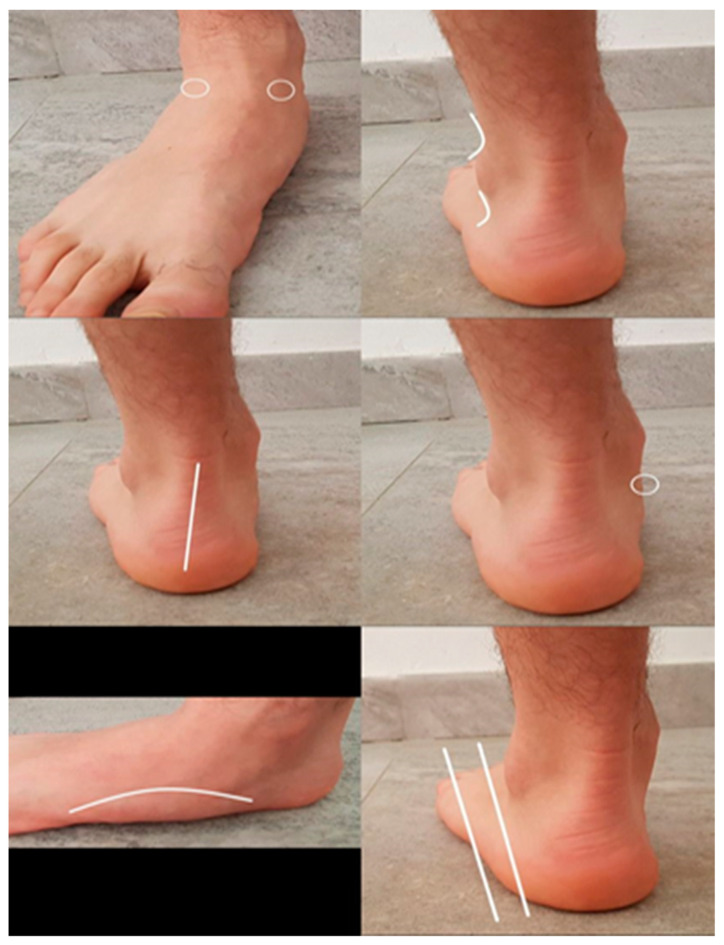
Identification of FPI criteria. The lines and circles in the figure indicate the points to be assessed in the FPI items.

The FPI and NDT variables were selected to assess the foot pronation, and their use is simple and easy to use. The FPI consists of six criteria, and to evaluate, the participant must be in a comfortable standing position where they support their feet as they usually do naturally. The evaluation lasted approximately two minutes, the maximum for each of the feet, and afterwards, an independent total value is obtained for each subject that indicates a prone, supinated, or normal posture that goes from −12 to + 12 values [19,27,28,29,30]. The six items are as follows: palpation of the talus, inframalleolar and supramalleolar curvature, position of the calcaneus in the frontal plane, prominence of the talo-scaphoid region, congruence of the medial longitudinal arch, and adduction or abduction of the forefoot (Figure 1). Each criterion is focused on obtaining the following classification on foot posture: a foot with a pronated position is the one that obtains a score greater than +6.

The NDT tool evaluates the difference between the original height of the navicular tuberosity in a sitting position without any weight load on the foot (first measurement), respect the second measurement when the drop of the scaphoid is produced when the body weight load falls on both feet and causes a collapse of the internal longitudinal foot arch, and, therefore, to the descent of the internal longitudinal arch. It is measured with a tape measure in millimetres [20]. The navicular drop test (NDT) is a simple clinical tool used to evaluate navicular bone descent as an indicator of the height of the medial arch of the foot in pronation. It presents a simple measurement protocol used in several studies in the field of sport, supporting its usefulness [31,32,33].

**Figure 2:**
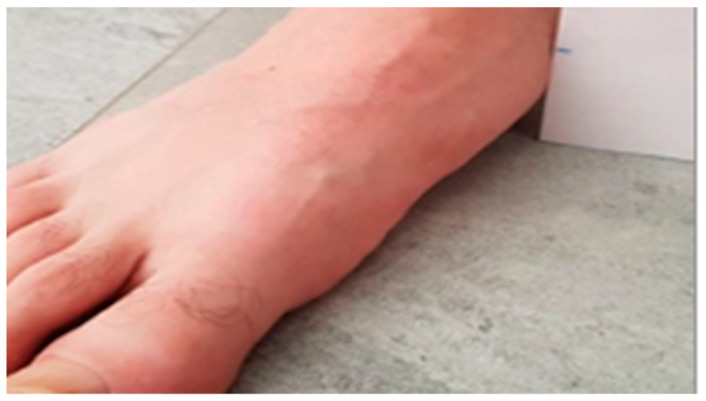
First measurement of the navicular NDT.

**Figure 3:**
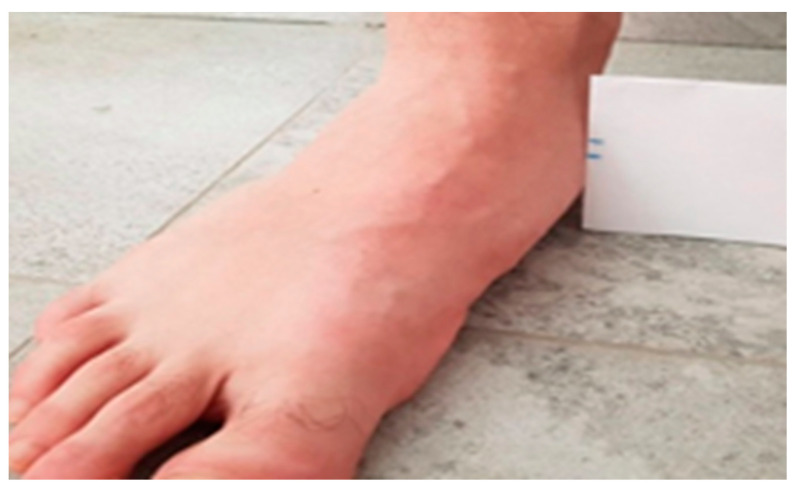
Second measurement after the descent of the navicular NDT.

The NDT is considered a clinical scientific tool with characteristics such as being easy to use that favour its use (simplicity, low cost, not requiring technology), thus highlighting the importance of its use in podiatric clinical practice and being considered as a reference value or normality (up to 10mm of descent of the navicular bone (Figure 2 and Figure 3)) [34].

Two days after baseline data (p1) was collected, the running or experimental activity was carried out for one hour, with the subject stopping at 30 min (p2), 45 min (p3), and 60 min (p4) to allow for evaluation of the FPI and NDT of both feet in each phase. The time was controlled by the same clock with a built-in alarm (Nixon Digital^®^, California, EE.UU.) for all subjects. Five days after the initial period (p1), the participants walked for one hour on the same equipment, stopping at the same periods p2, p3, and p4. In this case, it would be regarded as a control activity that would later be compared to a running activity. In all cases, the subject had to approach the place where the researcher was at the three predetermined times.

Both activities were carried out at Los Leones (Seville, Spain), a sports centre near the PCA, where there is an athletic track. In all cases, participants were told they could stop if they were tired or wanted to leave the study. In this study, the footwear that each participant wore in the activity was not controlled; each walked or ran according to his or her usual footwear for each modality and at a comfortable speed selected by them.

### 2.3. Statistical Analyses

**Figure 4:**
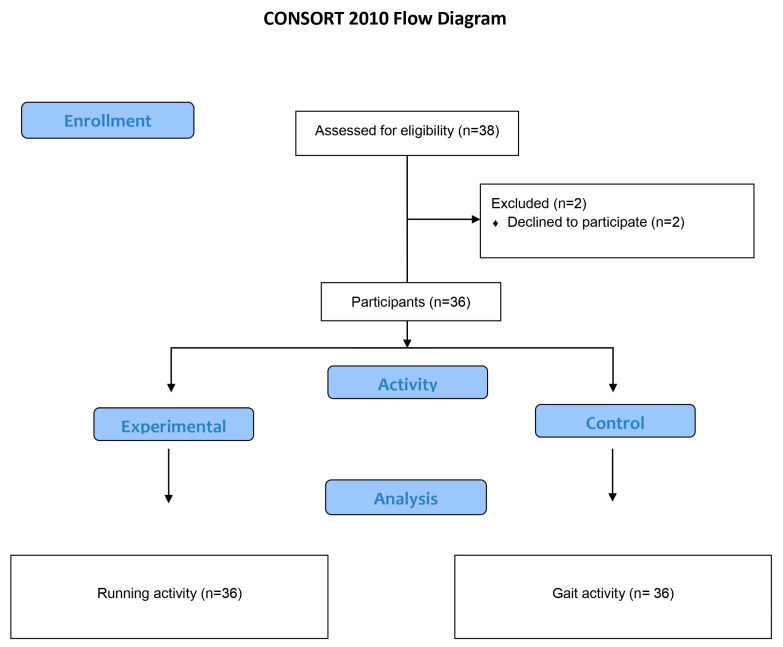
Prisma flow chart of the study.

The sample size of this study was designed to detect changes with an effect size greater than 0.8 (large effect size) for a contrast of means between two independent samples, assuming a type I error and a type II error of 0.05 and 0.2, respectively. Software G power 3.1.0 software (Franz Faul, Universität Kiel, Germany) [35] was used, resulting in a minimum sample size of 15 participants per group (30 feet per group). This size was increased to compensate for alterations in the statistical significance of the results caused by the possible dropout of participants. Only two subjects left the research (Figure 4).

For statistical analysis, the SPSS^®^ statistical software suite developed by IBM for data management version 26 for Windows program was used. The Shapiro–Wilk normality test and the Student’s t test of independent samples were used as a previous exploratory analysis. The Friedman test was used to evaluate the effects of the interventions and the differences in the primary outcomes p1 (baseline) for FPI and NDT and p2, p3, and p4 in both groups. The Wilcoxon signed-rank test was used to assess differences in p4 outcomes at 0 (baseline). Values of *p* ˂ 0.05 were considered statistically significant.

## 3. Results

A total of 17 women and 19 men participated in the study. The mean age was 33.4 ± 13.1 years, and the body mass indexes were situated at 22.6 ± 1.9 (the data is expressed as mean ± DS). The descriptive analysis of the FPI and NDT variables for foot amplifiers for each phase for the running group, as well as the intragroup analysis between the different phases with respect to the initial moment, is shown in Table 1.

Regarding the analysis of repeated measures for running activity, statistical significance is observed for both variables FPI and NDT when comparing the moments p1 and p3. No significance is observed when comparing p1 and p2, neither for the FPI variable nor for the NDT variable, which indicates that the significance occurs between the initial period and after physical activity of 45 min of running. The FPI showed a tendency to excessive pronation superior to the normal FPI value. The variables FPI and NDT for foot amplifiers for each phase for the walking group, as well as the intragroup analysis between the different phases with respect to the initial moment, are shown in Table 2.

Regarding the control or walking activity, statistical significance is observed when the said intragroup activity is analysed from the p3 period (45 min of activity) with respect to the FPI variable for both feet. While for the NDT variable, significance is obtained when comparing the initial period with the p4 period (60 min of activity). This indicates that the significance is shown for both feet after 45 min of walking for FPI and 60 min for NDT, and in both situations, the FPI and NDT maintain the normal value.

Inferential analysis of walking and running activity with respect to p2, p3, and p4 for both feet. When performing the inferential analysis between the two groups (running and walking), it is observed that there are significant differences with respect to FPI and NDT when comparing the period p1 to p2 and p3 to p4 for the right foot. With regard to the left foot, the significance appears when p1 is compared to the evaluation of p3 and p4 assessment with the FPI and for p2 in relation to NDT.

## 4. Discussion

This study compared a group of healthy recreational runners to determine whether, during a one-hour running period, muscle fatigue occurs and induces the foot to pronate more and to identify at what point in time this occurs (30, 45 or 60 min) with respect to the initial baseline situation (p1) compared to walking. The dependent variable, pronation, was assessed using the variables FPI and NDT, finding significant associations between the values of the navicular drop test and the foot posture index when comparing both activities in all cases after 60 min with respect to FPI and for both feet (p1 with respect to p4; *p* < 0.005).

The originality of this work was that pronation was evaluated in short time intervals or phases with respect to p1 or baseline and p2, p3, and p4 between the two groups. Data analysis showed that the FPI variable was significant for the right foot after 30 min of activity and for the left foot after 45 min, with FPI values indicating hyperpronation (FPI greater than +6) [19]. The NDT variable was significant after 30 min for both feet, showing an index of normality at this value, less than 10 mm. The results seem to show that running tends to modify the pronation of the foot toward pronation compared to walking.

Analysing the evaluation of the FPI variable between groups, our data are consistent with the results obtained by other studies [2,3,6,11,12,13,14,15,33,36,37,38,39]. One such study described a sample of 22 male runners with characteristics similar to those of this study showed a tendency to pronate the foot after a five km treadmill run. These results were consistent with our findings and similar and may indicate that after a distance travelled, the foot tends to pronation, which could be related to muscle fatigue. A decrease in MLA produced by foot pronation was measured by the dependent variables FPI and NDT. On the other hand, there was no control with another group and no assessment of the time spent in the activity. Other authors, such as Zuil-Escobar et al., observed that the mean navicular height decreased significantly after a half marathon (21 kilometres) by about 5 mm (*p* < 0.001), and the FPI-6 scores increased significantly after a half marathon (*p* < 0.001), defending the use of the variables used due to their good correlations when used in their studies (Spearman’s correlation coefficient = [0.663–0.703]). The FPI-6 scores increased significantly after a half marathon (*p* < 0.001) [38]. Again, the findings may be similar to those of this study, but the time factor was not considered in the distance recorded.

Most of these investigations have found a pronation tendency in the subjects. It is important to note that the sample studied here consisted of non-pronators. Regarding the influence of leg dominance that results in differences in lower limb kinematics and kinetics between the two feet, in this study, it was observed that the behaviour with respect to FPI in the left foot was not equal with respect to time in the right foot (p2 significant with respect to p1). However, this is speculative, and the dominance of the legs was not recorded in the current study or others described [12,37].

Most studies can be seen to justify the tendency to pronation in relation to muscle fatigue that affects the morphology of MLA, which collapses mainly due to the action of the intrinsic musculature [39]. This fact was evidenced by Okamura et al., who determined the kinematic patterns of the foot during running in runners with pronated feet and established a relationship between these patterns and the morphology of the intrinsic musculature of the foot [5,6] after evaluating the abductor hallucis, the hallucis brevis and longus flexor, the digitorum brevis and longus flexor, and the longus peroneus muscles. These authors studied 21 runners with pronated feet using the NDT variable in relation to these muscle groups, concluding that the kinematics of the foot during running were affected by the morphology and function of the intrinsic medial foot muscles [6].

A recent systematic review found limited evidence regarding changes in kinematics and kinetics during strenuous running in relation to performance and injury. They justified the need for higher quality studies, with a larger sample of homogeneous runners and a carefully selected type of running to provide quality information for injury prevention [15].

The literature also points to a possible relationship between scaphoid drop during gait in healthy subjects and increased walking speed during gait [40]. These data showed the correlation that higher speed produced a more significant drop in scaphoid height. When subjects walked and ran at the same speed, there was an increase in dynamic scaphoid drop greater than 3.5 mm (*p* < 0.001) in running [40]. This parameter has not been controlled in our work but could be a factor to consider. These findings highlight the need for a specific task during the NDT gait analysis in relation to possible differences between running and walking and even refer to the possibility of differences between running and over-ground walking [40].

These findings seem to indicate that biomechanical changes occur in the locomotor apparatus, which, as mentioned above, can affect the knee, back, ankle, and foot [38,41].

Consequently, it seems that our results could be justified as fatigue of the foot musculature and showed a coherent tendency with respect to the work presented, although there is no work that has analysed this tendency in such short time intervals in healthy subjects. Running has generally been observed to tend to cause foot hyperpronation after 45 min of activity for both feet, which may predispose to injury in other parts of the body.

However, intrinsic muscle strengthening has been of recent interest and should be considered a conservative treatment for runners who are believed to have weak intrinsic muscles in relation to injuries in other parts of the body. According to the scientific literature, foot disorders are defended in relation to the risk of injury. It is relevant to consider that the subjects were allowed to modulate their more comfortable and economical walking speed; for this reason, this work has allowed each person to walk or run at their cruising speed. We considered that this could be a bias if we did not allow for the most appropriate speed decided by each participant.

Limitations of the study were as follows: the sample size of this study was small, and the laterality and assessment of the intrinsic foot musculature were not considered and can be considered a limitation. In this research, the subjects modulated their more comfortable and economical walking speed, and for this reason, this work has allowed each person to walk or run at their cruising speed.

The practical implications of the study could help identify excessive foot pronation or hyperpronation that may be induced by muscle fatigue. Knowledge of factors that may affect foot disorders can help develop personalised training based on the needs of each subject, can increase the level of health of the population as well as prevent injuries in running sports training.

## 5. Conclusions

The sample of this research composed of recreational non-pronator runners showed a tendency to foot pronation after 45 min of running activity compared to normal walking measured by the FPI and at 30 min as assessed by the NDT. Running activity induces a hyperpronation foot posture index after 30 min of running for the right foot and for the left foot after 45 min. The running activity shows a major tendency toward foot pronation, with regards to the same normal walking period.

Future research is necessary.

## Figures and Tables

**Table 1:** FPI and NDT variables for the right and left foot in each period. The data are expressed for the running activity (mean, standard deviation, median, and interquartile range) and significance.

n = 72				Running			*p* Value
	Period	Mean	DS	Median	RIQ	p1–p3	p1–p4
Right FPI	p1	2.6	2.5	2.5	2.0–3.0	<0.001 *	<0.001 *
	p2	4.1	2.4	4.0	2.0–5.0		
	p3	6.2	2.8	7.0	5.0–7.0		
	p4	7.9	2.8	9.0	7.0–10.0		
Left FPI	p1	2.6	2.6	3.0	1.0–4.0	<0.001 *	<0.001 *
	p2	3.6	2.9	4.0	2.0–5.0		
	p3	5.4	2.0	5.0	5.0–7.0		
	p4	7.1	2.4	7.0	6.0–8.0		
Right NDT	p1	4.4	1.7	4.5	3.0–5.0	<0.001 *	<0.001 *
	p2	5.6	1.9	5.0	5.0–6.0		
	p3	6.7	1.7	6.0	6.0–8.0		
	p4	7.8	2.0	7.0	7.0–9.0		
Left NDT	p1	4.4	1.8	4.0	3.0–5.0	<0.001 *	<0.001 *
	p2	5.4	1.7	5.0	5.0–6.0		
	p3	6.4	1.6	6.0	5.0–7.0		
	p4	7.6	2.1	7.0	6.0–9.0		

Friedman test. Signification level * *p* < 0.001; RIQ: Interquartile range. DS: Standard deviation.

**Table 2:** FPI and NDT variables for the right and left feet in each period. Data are expressed for walking activity (mean, standard deviation, median, and interquartile range) and significance.

n = 72				Walking		*p* Value	
		Mean	D.S	Median	RIQ	p1–p3	p1–p4
Right FPI	p1	2.6	2.5	2.5	2–3	0.002 *	<0.001 *
	p2	2.9	2.5	3	2–4		
	p3	3.9	2.4	4	3–4		
	p4	4.8	2.6	5	4–5		
Left FPI	p1	2.6	2.6	3	1–4	0.001 *	<0.001 *
	p2	2.8	2.7	3	1–4		
	p3	3.7	3.0	3	3–5		
	p4	4.3	2.9	4	3–5		
Right NDT	p1	4.4	1.7	4.5	3–5		<0.001 *
	p2	4.5	1.9	5	3–5		
	p3	4.7	1.9	5	3–5		
	p4	5.7	2.1	6	5–6		
Left NDT	p1	4.4	1.9	4	3–5		<0.001 *
	p2	4.4	2.0	4	3–5		
	p3	4.7	2.1	4	3–6		
	p4	5.2	2.1	5	3–6		

Friedman test. Signification level * *p* <0.001. RIQ: Interquartile range. DS: Standard deviation.

## Data Availability

Data are contained within the article.
